# Natural soda lakes provide compatible conditions for RNA and membrane function that could have enabled the origin of life

**DOI:** 10.1093/pnasnexus/pgae084

**Published:** 2024-03-19

**Authors:** Zachary R Cohen, Dian Ding, Lijun Zhou, Saurja DasGupta, Sebastian Haas, Kimberly P Sinclair, Zoe R Todd, Roy A Black, Jack W Szostak, David C Catling

**Affiliations:** Department of Chemistry, University of Washington, Seattle, WA 98195, USA; Astrobiology Program, University of Washington, Seattle, WA 98195, USA; Department of Chemistry and Chemical Biology, Harvard University, Cambridge, MA 02138, USA; Department of Molecular Biology and Center for Computational and Integrative Biology, Massachusetts General Hospital, Boston, MA 02114, USA; Department of Biochemistry and Biophysics and Penn Institute for RNA Innovation, Perelman School of Medicine, University of Pennsylvania, Philadelphia, PA 19104, USA; Department of Chemistry and Biochemistry, University of Notre Dame, Notre Dame, IN 46556, USA; Astrobiology Program, University of Washington, Seattle, WA 98195, USA; Department of Earth and Space Sciences, University of Washington, Seattle, WA 98195, USA; Astrobiology Program, University of Washington, Seattle, WA 98195, USA; Department of Earth and Space Sciences, University of Washington, Seattle, WA 98195, USA; Astrobiology Program, University of Washington, Seattle, WA 98195, USA; Department of Earth and Space Sciences, University of Washington, Seattle, WA 98195, USA; Department of Chemistry and Department of Astronomy, University of Wisconsin, Madison, WI 53706, USA; Department of Chemistry, University of Washington, Seattle, WA 98195, USA; Astrobiology Program, University of Washington, Seattle, WA 98195, USA; Howard Hughes Medical Institute, Department of Chemistry, University of Chicago, Chicago, IL 60637, USA; Astrobiology Program, University of Washington, Seattle, WA 98195, USA; Department of Earth and Space Sciences, University of Washington, Seattle, WA 98195, USA

**Keywords:** protocell, RNA, ribozyme, membrane

## Abstract

The origin of life likely occurred within environments that concentrated cellular precursors and enabled their co-assembly into cells. Soda lakes (those dominated by Na^+^ ions and carbonate species) can concentrate precursors of RNA and membranes, such as phosphate, cyanide, and fatty acids. Subsequent assembly of RNA and membranes into cells is a long-standing problem because RNA function requires divalent cations, e.g. Mg^2+^, but Mg^2+^ disrupts fatty acid membranes. The low solubility of Mg-containing carbonates limits soda lakes to moderate Mg^2+^ concentrations (∼1 mM), so we investigated whether both RNAs and membranes function within these lakes. We collected water from Last Chance Lake and Goodenough Lake in Canada. Because we sampled after seasonal evaporation, the lake water contained ∼1 M Na^+^ and ∼1 mM Mg^2+^ near pH 10. In the laboratory, nonenzymatic, RNA-templated polymerization of 2-aminoimidazole-activated ribonucleotides occurred at comparable rates in lake water and standard laboratory conditions (50 mM MgCl_2_, pH 8). Additionally, we found that a ligase ribozyme that uses oligonucleotide substrates activated with 2-aminoimidazole was active in lake water after adjusting pH from ∼10 to 9. We also observed that decanoic acid and decanol assembled into vesicles in a dilute solution that resembled lake water after seasonal rains, and that those vesicles retained encapsulated solutes despite salt-induced flocculation when the external solution was replaced with dry-season lake water. By identifying compatible conditions for nonenzymatic and ribozyme-catalyzed RNA assembly, and for encapsulation by membranes, our results suggest that soda lakes could have enabled cellular life to emerge on Earth, and perhaps elsewhere.

**Significance Statement**

Where did cells originate on the early Earth? The first cells (protocells) are thought to have consisted of informational and catalytic RNAs inside membrane vesicles. However, RNA function requires divalent cations, such as Mg^2+^, whereas membranes of environmentally available amphiphiles (e.g. fatty acids) are disrupted by divalent cations. Here, we show that natural soda lake water, which contains ∼1 mM divalent cations, could provide a suitable environment for three processes likely important for the origin of cellular life: nonenzymatic RNA polymerization, ribozyme activity, and encapsulation by prebiotic membranes. Our results suggest that soda lakes deserve further study as potential environments for the formation of the first cells.

## Introduction

An early stage of life on Earth may have consisted of protocells that had genetic molecules encapsulated by lipid membranes (1). RNA may have served an important role in protocells because it can store and transmit genetic information and catalyze reactions (2). Protocell membranes may have been composed of fatty acids, which enable the exchange of small molecules like RNA building blocks and metabolites with the external environment while encapsulating large polymers (3). However, typical concentrations of divalent cations like Mg^2+^ that promote nonenzymatic RNA copying (4) and ribozyme activity (5) disrupt fatty acid membranes, leading to the release of encapsulated solutes (6). Chelation of divalent cations by excess citrate preserves fatty acid vesicles and enables internal RNA reactions (7), but high concentrations of citrate on the early Earth are unlikely. Serine and glycine maintain the integrity of fatty acid vesicles in 10 mM Mg^2+^ by increasing lamellarity (8), but the assembly of RNA has not been investigated inside these vesicles. Interestingly, nonenzymatic synthesis of a genetic polymer similar to RNA (NP-DNA (3)) and ribozyme activity (9, 10) have both been observed within fatty acid vesicles when the concentration of divalent cations is below 4 mM.

**Fig. 1.**
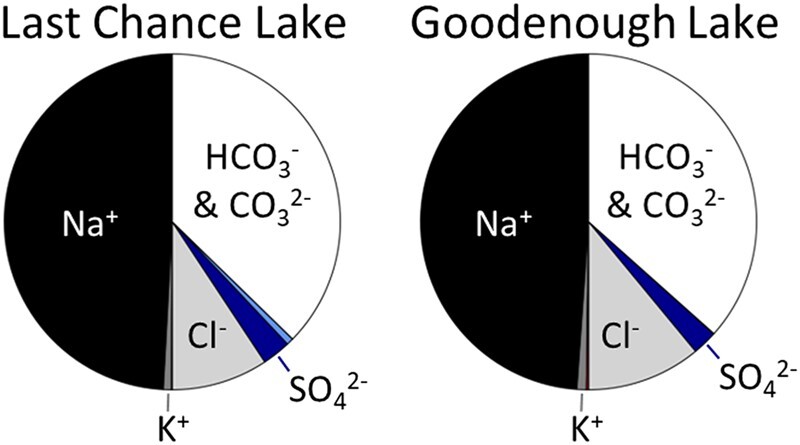
Composition of soda lake waters used in experiments, illustrating their dominant sodium–carbonate–chloride composition (Eq/L = molar concentration × charge). Positive charge on the left of each pie chart is balanced by negative charge on the right. The data are for single measurements of water samples collected in November 2021. All concentrations, including smaller components <10 mM, such as Mg^2+^, Ca^2+^, and PO_4_^3−^ (measured by Haas et al. (27)), are shown in Tables S1–S3.

Soda lakes, in which Na^+^ and (bi)carbonate are the dominant dissolved species (Fig. 1), are promising sites for the co-assembly of RNA and fatty acids into protocells. These lakes contain 0.1–1 M of bicarbonate and carbonate anions (11). Because salts of divalent cations and carbonate anions, i.e. (Ca, Mg, Fe)CO_3_, have poor solubility, the concentration of divalent cations in soda lakes is on the order of ∼1 mM (12). Additionally, soda lakes on the early Earth may have concentrated phosphate (13), ferrocyanide (14), and sulfur species (15), potentially enabling the synthesis of ribonucleotides during photochemically driven reductive homologation of hydrogen cyanide (16, 17). Fatty acids could have been delivered to Earth by meteorites (18), synthesized endogenously on metal surfaces (19), or synthesized during electrochemical sparking (20), and those fatty acids could have been subsequently concentrated in soda lakes via evaporation to enable membrane formation (21). Although there is uncertainty about how much subaerial continental crust was present on the early Earth (22), zircon geochemistry shows that continental crust was present from ∼4.4 Gya and plausibly consistent with surface exposure (23). Also, volcanic hotspot islands and extensive plateaus are expected (24), where the former are sites of soda lakes today (25). Indeed, modern soda lakes are common on volcanic terrain (26).

Because soda lakes provide unique opportunities for the prebiotic formation of RNA and fatty acid vesicles, and because relatively low concentrations of divalent cations have been shown to enable RNA assembly within fatty acid vesicles, we investigated key components of protocell assembly within natural soda lake water. We collected water samples from Last Chance Lake and Goodenough Lake on the Cariboo Plateau in British Columbia, Canada, in November 2021. These soda lakes have some of the highest soluble phosphate concentrations of lakes on Earth (27), with Last Chance Lake reaching the highest of all. We collected after summertime evaporation when these lakes each contained ∼1 M Na^+^ and ∼1 mM Mg^2+^ at pH 10 (Tables S1–S3). We tested for nonenzymatic, RNA-templated synthesis of RNA in water from both soda lakes. Additionally, we established the activity of a ligase ribozyme in natural lake water, and we tested whether membranes composed of fatty acids and fatty alcohols can encapsulate solutes in natural lake water. Our findings directly inform the search for natural environments that could have supported the origin of cellular life on Earth.

## Results and discussion

### Nonenzymatic, RNA-templated synthesis of RNA

**Fig. 2.**
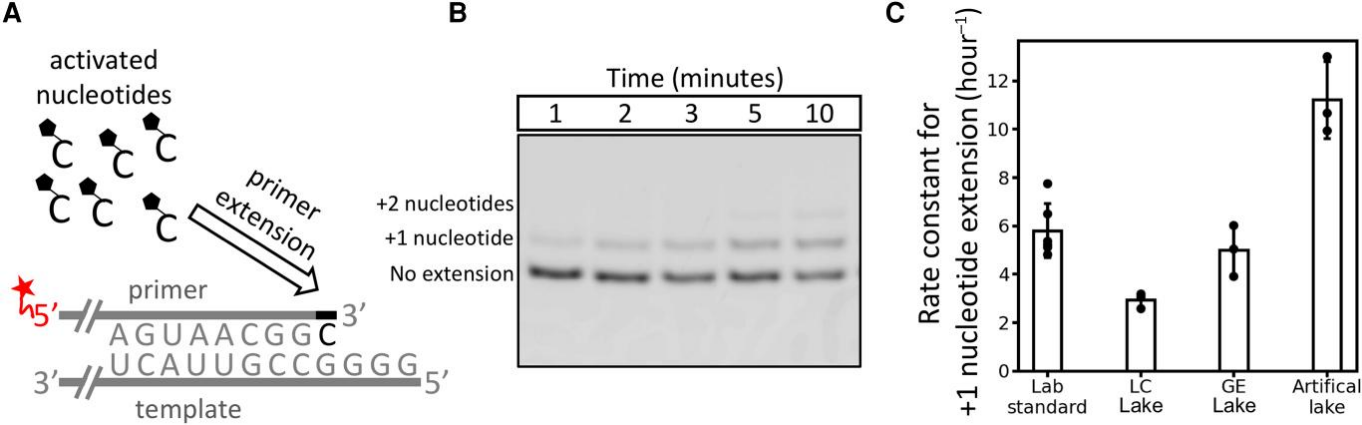
RNA primers on RNA templates are extended at comparable rates in soda lake water and in standard laboratory conditions. A) Schematic of RNA primer extension by 2-aminoimidazole-activated ribonucleotides. Our data suggest that primer extension in lake water proceeds through a bridged dinucleotide intermediate (Fig. S3), which is consistent with previous results (31). The primer is fluorescently labeled at the 5′ end. B) Gel image showing RNA-templated extension of an RNA primer in Last Chance Lake water within 10 min. RNAs smaller than the primer were not detected, suggesting that the RNA primer is not hydrolyzed. C) First-order rate constants for extension of an RNA primer by one nucleotide. Standard laboratory conditions are 50 mM MgCl_2_ and 0.2 M bis-tris-propane at pH 8, and artificial lake water is 0.5 M Na_2_CO_3_, 1 mM MgCl_2_, and 0.2 M bis-tris-propane at pH 10. Error bars correspond to the SD from three independent experiments in lake waters, and six independent experiments in standard laboratory conditions. LC is Last Chance Lake and GE is Goodenough Lake.

Before enzymes emerged on the early Earth, short RNAs that formed during wet-dry cycling (28), freeze-thaw cycling (29), or on mineral surfaces (30) may have been replicated by successive additions of chemically activated ribonucleotides. For example, after pairing between a short RNA primer and the template, 2-aminoimidazole-activated nucleotides can base pair with the template and react nonenzymatically with the primer to form a 3′-5′ phosphodiester linkage (Fig. 2A). Unlike polymerization of triphosphate nucleotides, 2-aminoimidazole-activated nucleotides spontaneously form 5′-5′ bridged dinucleotide intermediates that enable rapid, nonenzymatic extension of RNA primers (31). Separation of the strands may enable a new cycle of polymerization to generate a copy of the original template. Nonenzymatic primer extension may have been a necessary step in the formation of the first ribozymes, so we investigated whether this process can proceed in soda lake water.

We found that the rates of primer extension in lake water were comparable with the rates in standard laboratory conditions (Fig. 2). We carried out reactions in 90% natural water from Last Chance Lake (LC) or Goodenough Lake (GE), and we also prepared the reaction in artificial lake water that approximated the cation composition, anion composition, and pH of the natural lakes (0.5 M Na_2_CO_3_, 1 mM MgCl_2_, and 200 mM bis-tris-propane at pH 10). Standard laboratory conditions were chosen to be 50 mM MgCl_2_ and 200 mM bis-tris-propane at pH 8, consistent with recent work on nonenzymatic primer extension (32–34). We used 2-aminoimidazole-activated cytidine monophosphate nucleotides to extend the 3′ end of primers on a template with four unpaired guanosine nucleotides. We measured the relative abundance of fluorescently labeled primers over time and calculated pseudo first-order rate constants for +1 nucleotide extension in each set of conditions (Fig. S1).

The surprisingly fast initial rate of primer extension may be explained by the relatively high pH of the lake water. In artificial solutions that approximate the cation composition of natural lakes, the rate of primer extension increased rapidly with pH (Fig. S2). As pH increases, the extent of deprotonation of the primer 3′ hydroxyl also increases, unleashing a more potent nucleophile and enhancing the rate of primer extension (35). Rapid primer extension in natural lake water also relies on the formation of a bridged dinucleotide intermediate, the same as the mechanism for primer extension in laboratory conditions (31). When we prevented the formation of the bridged dinucleotide by supplying an excess of 2-aminoimidazole, the yield of extended primers in lake water was greatly reduced (Fig. S3). However, in the absence of primer and template RNAs, we did not observe the formation of the bridged dinucleotide in natural lake water by ^31^P NMR (Fig. S4). Although trace amounts of bridged dinucleotide could have been supplied with the activated mononucleotides, we cannot detect an obvious bias in the initial rate of primer extension: in both lake samples and standard laboratory conditions, there is an initial ∼5 min period where the rate of primer extension is maximum, followed by sustained extension at a lower rate (Fig. S5).

**Fig. 3.**
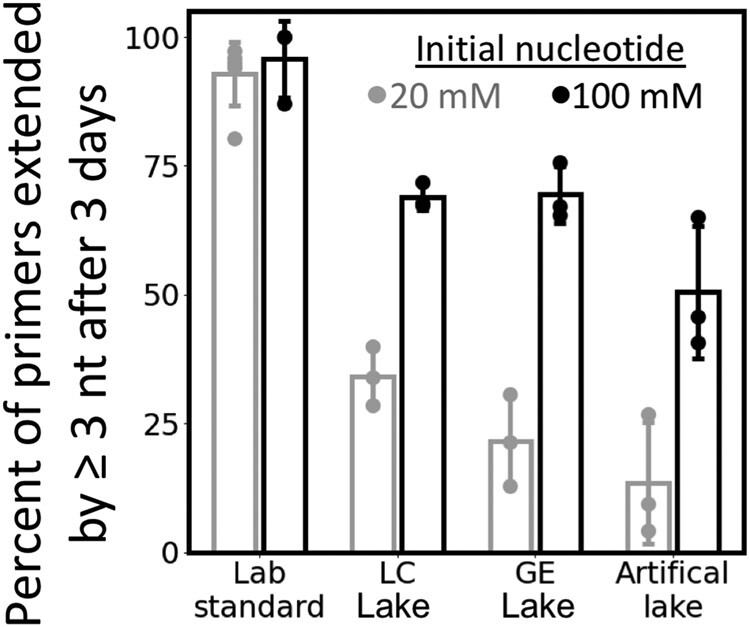
The yield of extended RNA primers is lower in soda lake water than standard laboratory conditions, although the yield in lake water increases by supplying a larger initial concentration of activated nucleotides (nt). The data show the fraction of RNA primers that were extended by at least three nucleotides in natural and artificial lake water after 3 days, when either 20 mM (gray) or 100 mM (black) activated nucleotides is supplied. We did not observe any hydrolysis of the primer after 3 days (Fig. S6). Standard laboratory conditions are 50 mM MgCl_2_ and 200 mM bis-tris-propane at pH 8, and artificial lake water is 0.5 M Na_2_CO_3_, 1 mM MgCl_2_, and 200 mM bis-tris-propane at pH 10. Error bars correspond to the SD from three independent experiments, except for the error bars for the 20 mM initial nucleotide sample in laboratory standard conditions which correspond to six independent experiments. LC is Last Chance Lake and GE is Goodenough Lake.

We did not observe hydrolysis of RNA primers after 72 hours in natural or artificial lake water (Fig. S6), which is roughly consistent with previous estimates of ∼1% RNA degradation after 72 hours in 1 M K^+^ and 5 mM Mg^2+^ at pH 9.5 (36). The relatively low concentration of divalent cations may compensate for the high pH in lake water to keep the hydrolysis rate low (37). However, we also observed that fewer primers had been extended by at least three nucleotides after 72 hours in lake water, relative to standard laboratory conditions (Fig. 3). Why are fewer primers extended after 72 hours in lake water than in laboratory conditions, despite comparable initial rates of extension? Carbonate anions in the lake water seem to play a role; artificial lake solutions that approximate the cation composition and pH of natural lakes showed lower yields of extended primers after 72 hours—but comparable initial rates of primer extension—when carbonate is the most abundant anion (Fig. S7). The high pH of carbonate lake water is likely to have had negative effects on the yields of extended primers after 72 hours: fewer bridged dinucleotide intermediates form when the pH is high, and these intermediates will not bind as strongly to the template due to deprotonation of the G and U nucleobases. Although these factors probably contributed to the low yields of extended primers after 3 days in lake water, it is encouraging that the initial rates of primer extension were not slowed as severely.

After increasing the initial concentration of activated nucleotides from 20 to 100 mM, we observed more primer extension after 72 hours in lake water (Fig. 3). The percentage of primers that had been extended by three nucleotides nearly doubled in all lake samples due to the higher initial concentration of nucleotides. Although fewer primers were extended in lake water than in laboratory conditions per activated nucleotide that was supplied, our observations show that considerable primer extension is possible in natural soda lakes when the concentration of activated nucleotides is relatively high. High concentrations of activated nucleotides could have been achieved through the evaporation of shallow soda lakes on the early Earth, while maintaining ∼1 mM divalent cations because of the poor solubility of Mg-containing carbonates (13). Last Chance Lake, for example, was observed to be a dry salt flat in September 2022 with some water beneath salt crusts (27). Continuous activation of nucleotides in situ (38) or replenishment in a natural flow reactor could have improved the yields of extended primers. The presence of 5′-5′ mononucleotide-bridged-oligonucleotides could further improve primer extension in natural lakes because these substrates bind more tightly to RNA templates, enabling rapid primer extension (39, 40).

We note that full extension of the primer on the template (i.e. +4 nucleotides) is exceedingly slow (Fig. S8). This problem of “last nucleotide addition” has previously been observed in laboratory conditions because dinucleotide intermediates can only form 1 base pair with the template's terminal nucleotide, leading to poor binding affinity of the substrate (41). However, protocells may not have needed to synthesize the full complementary strand for a given template. Unlike modern cells that contain large linear or circular genomes, protocells may have contained small, virtual circular genomes where RNA synthesis can be transiently templated by multiple oligomers at different positions for replication (42, 43). Additionally, we recognize that 72 hours is not an endpoint for nonenzymatic primer extension in lake water. We observed sustained primer extension beyond 72 hours, which would further improve the yield of extended primers (Fig. S9). However, we did observe partial hydrolysis of the primer after 6 days (Fig. S6). Although elevated temperatures lead to faster RNA hydrolysis, recent simulations suggest that the average surface temperature of the Earth would have been near 0 °C from about 4.4 to 4.0 billion years ago, when life likely originated (44). Taken together, our findings suggest that nonenzymatic primer extension could proceed robustly in natural soda lakes, especially if the lake pH is somewhat lower, which is to be expected under an early Earth atmosphere with a higher CO_2_ concentration (14, 44).

### Activity of a ligase ribozyme

Sustained nonenzymatic replication could enable the evolution of ribozymes, which could then catalyze RNA replication. Specifically, ligase ribozymes could enable RNA replication by joining small RNAs together on a template. We previously identified by in vitro selection a 70-nucleotide ribozyme that catalyzes ligation between its 3′ end and a substrate RNA activated by 2-aminoimidazole in the presence of Mg^2+^ concentrations as low as 1 mM (10, 45). Therefore, we conducted experiments to determine whether this ligase ribozyme functions in natural soda lake water.

**Fig. 4.**
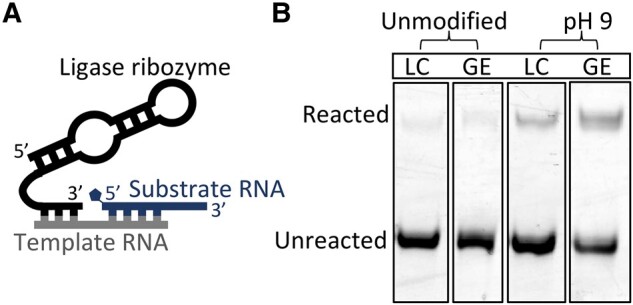
A ligase ribozyme is active in soda lake water after decreasing pH from ∼10 to 9, as might occur under the higher atmospheric pCO_2_ of the early Earth. A) The ribozyme and substrate base pair to the same RNA template. The 5′ end of the substrate is activated with 2-aminoimidazole, and the ribozyme ligates the substrate to its 3′ end. B) After 20 hours in unmodified water (pH ∼10) from either Last Chance Lake (LC) or Goodenough Lake (GE), the ribozyme activity is low. When the lake water was adjusted to pH 9 by bubbling in carbon dioxide, the ribozyme activity increased substantially. In three replicate experiments, RNA-catalyzed ligation yields were 11 ± 1 and 32 ± 3% after 20 hours in pH 9 lake water from LC or GE, respectively (Fig. S10).

We observed low activity of the ligase ribozyme in 70% natural water from Last Chance Lake and Goodenough Lake; however, the ribozyme became active when the lake water was modified by decreasing the pH from ∼10 to 9 by bubbling in carbon dioxide (Fig. 4). This pH sensitivity is not surprising: the ribozyme was selected at pH 8, so it is reasonable to expect higher activity as the solution becomes more like the selection conditions. We observed RNA-catalyzed ligation yields of 32 ± 3% in pH 9–adjusted water from Goodenough Lake, and 11 ± 1% in pH 9–adjusted water from Last Chance Lake after 20 hours. The ribozyme activity in lake water was lower than the activity in laboratory conditions (0.362 M NaCl, 1.2 mM MgCl_2_, and 242 mM bis-tris-propane at pH 8) where we observed a ligation yield of 60 ± 6% after 20 hours (Fig. S10). When the ribozyme was replaced with an inactive RNA containing a completely randomized catalytic domain sequence, we did not observe ligation under any conditions (Fig. S11).

We performed experiments with artificial solutions to validate our results from the natural lake water. We consistently observed that decreasing the pH below 10 leads to higher ribozyme activity, whether the ionic composition mimics the natural lake (∼0.5 M Na_2_CO_3_ and ∼1 mM MgCl_2_) or laboratory conditions (Fig. S12). Additionally, we observed that a high concentration of monovalent cations, which are present in the natural lake water, leads to lower yields of ribozyme-catalyzed ligation (10). In general, our data demonstrate that ribozymes can function in soda lake water, though it is clear that our chosen ribozyme is not particularly well adapted to soda lake water. We expect that selection in soda lake water would yield ribozymes with much higher activity under these conditions of high pH and salinity, and that natural selection in soda lake water could have generated diverse ribozymes during the origin of life.

It is plausible that soda lakes had a lower pH on the early Earth (between pH 6.5 and 9). Coupled carbon cycle—climate simulations show that atmospheric CO_2_ is generally expected to have been higher by ∼1 to 3 orders of magnitude before 4 billion years ago (44). Under such conditions, the pH of lake water is lowered (14), as in our experiments.

### Encapsulation by prebiotic membranes

To determine whether prebiotically plausible amphiphiles can assemble into vesicles in water from soda lakes during the dry season after seasonal evaporation, we prepared a solution of 89% Last Chance Lake water containing 112.5 mM decanoic acid and 112.5 mM decanol with 0.1 mM carboxyfluorescein. The amphiphiles immediately floated to the surface, which is consistent with the behavior of flocculated vesicles in saturated solutions of monovalent cations (46). We replaced the exterior solution around the amphiphiles with 100% water from Last Chance Lake in order to remove unencapsulated carboxyfluorescein (Fig. S13). However, we did not observe retention of internal carboxyfluorescein, suggesting that the amphiphiles did not encapsulate the solution (Fig. S14). We subsequently diluted the salt concentration, and we did not observe separated vesicles with encapsulated carboxyfluorescein (Fig. S14). We conclude that the formation of vesicles capable of encapsulating solutions is inefficient in natural water during Last Chance Lake's dry season.

**Fig. 5.**
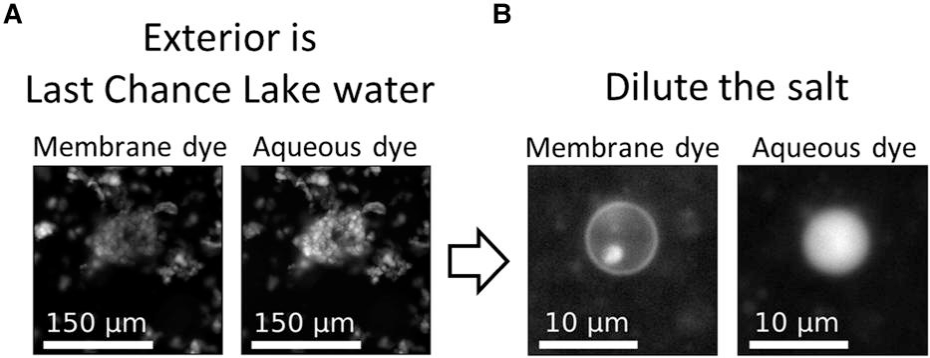
Vesicles of 1:1 decanoic acid:decanol encapsulate aqueous carboxyfluorescein dye for >1 hour when the exterior solution is water from Last Chance Lake. A) Monovalent salts induce the flocculation of vesicles into large aggregates. Vesicles were initially assembled in a dilute salt solution of 0.1 M NaHCO_3_ at pH 10 with 0.1 mM carboxyfluorescein dye before the exterior solution was replaced with Last Chance Lake water (Fig. S13). B) Subsequent dilution into a 56-mM decanoic acid and 0.1 M NaHCO_3_, pH 10 solution causes vesicles to separate from each other, and separated vesicles still retain encapsulated dye. Similar results were obtained for vesicles in artificial lake water; however, we could not determine whether vesicles retained encapsulated dye in water from Goodenough Lake (Fig. S17). Rhodamine 6G membrane dye was added immediately prior to imaging. Full field images are in Fig. S15.

Given that our water sample is from Last Chance Lake's dry season, we tested whether amphiphiles encapsulate solutes if they assemble into vesicles in dilute solution during the lake's wet season or after rainfall events, and if those vesicles could retain encapsulated solutes as the exterior solution becomes saltier during evaporation in the dry season. We assembled vesicles of 1:1 decanoic acid:decanol in a relatively dilute salt solution of 0.1 M NaHCO_3_ at pH 10 with 0.1 mM carboxyfluorescein. These conditions mimic dilute lake water during the wet season. We replaced the exterior solution around the vesicles with Last Chance Lake water from the dry season containing ∼1 M Na^+^ and ∼1 mM Mg^2+^ at pH 10, and we imaged the vesicles with fluorescence microscopy (Fig. 5). We observed flocculated vesicles that retain encapsulated carboxyfluorescein despite the millimolar concentration of divalent cations in the surrounding natural lake water. Excellent contrast between the encapsulated carboxyfluorescein and the exterior solution indicated that the flocculated vesicles were surrounded by Last Chance Lake water. We subsequently diluted the salt concentration in the exterior solution to ∼100 mM NaHCO_3_ and found that separated vesicles still retained the dye. Full field images (Fig. S15) and a size-exclusion chromatogram (Fig. S14) show that many vesicles retain dye after dilution of the salt. Our results in Last Chance Lake water are consistent with the behavior of flocculated vesicles in saturated solutions of monovalent cations, where we observed retention of encapsulated carboxyfluorescein for at least 3 hours (46).

We also found that vesicles retained encapsulated dye when the exterior solution is artificial lake water of 0.75 M Na_2_CO_3_ and 1 mM MgCl_2_ at pH 10, or artificial lake water of 1 M NaCl, 1 mM MgCl_2_, and 0.1 M NaHCO_3_ at pH 10 (Fig. S16). However, because our method of exchanging the exterior solution around vesicles requires an extremely high concentration of unchelated monovalent cations, we were unable to determine whether vesicles retain encapsulated dye in the less Na^+^-rich water from Goodenough Lake or in artificial lake water of 0.5 M Na_2_CO_3_ and 1 mM MgCl_2_ at pH 10 (Fig. S17). In summary, our results show that vesicles can encapsulate solutes in soda lake water after seasonal evaporation if the vesicles are allowed to assemble in a dilute solution during the wet season, or after a rainfall event in an otherwise dry season.

## Conclusion

Our results suggest that soda lakes on the early Earth could have supported key features of protocell development. The nonenzymatic synthesis of RNA proceeds on RNA templates at a comparable rate in soda lake water as under standard laboratory conditions. We find that the yield of nonenzymatic RNA polymerization increases with the concentration of activated nucleotides. Activated nucleotides could have reached high concentrations during evaporation from natural lakes, although continued nucleotide synthesis may become limited by the availability of phosphate [∼40 mM in Last Chance Lake, but potentially higher in prebiotic lakes (13, 27)]. 2-Aminoimidazole could be produced from nucleotide precursors (47), and nucleotide activation with 2-aminoimidazole could occur in the eutectic brines of partially frozen lakes (34). While nonenzymatic copying of sequence-general RNA templates remains challenging (48), our results show that soda lakes are capable of supporting nonenzymatic RNA synthesis in a comparable manner to optimized laboratory conditions.

Sustained nonenzymatic replication could have enabled the evolution of ribozymes, which could then catalyze RNA replication, a key step during the origin of cells. Ligase ribozymes could facilitate RNA-catalyzed RNA replication, and the use of a 2-aminoimidazole-activated substrate could have enabled a continuous path from nonenzymatic to ribozyme-catalyzed RNA replication. We find that our in vitro selected ligase ribozyme retains activity in soda lake water when the pH is adjusted to resemble the in vitro selection conditions. Ribozymes that evolved in natural lakes would be optimized to function under such conditions, and these ribozymes could be isolated in the laboratory by performing in vitro selection experiments in natural lake water. In fact, a previous in vitro evolution experiment yielded ribozymes with ligase activity at pH 9.8 from an initial population that was inactive at this pH (49). Additionally, soda lakes on the early Earth may have had a lower pH (between pH 6.5 and 9) due to a high concentration of CO_2_ in the atmosphere (13, 44).

Additionally, we show that prebiotic membranes can encapsulate solutes despite flocculation in soda lake water. Although the internal volume of vesicles may decrease as the external salt concentration increases, our results show that material can remain encapsulated after the vesicles swell again following dilution of the salt. It is likely that nonenzymatic RNA synthesis (50) and ribozyme-catalyzed ligation (10) can proceed inside flocculated vesicles whose exterior solution is soda lake water. However, future work is needed to identify conditions where 2-aminoimidazole-activated mononucleotides or oligonucleotides can permeate into flocculated vesicles without releasing larger RNAs. We envision that RNA–membrane interactions could modulate both the extent of flocculation and the permeability of the flocculated vesicles (51). Fatty acid vesicles have been shown to form in other putative early Earth environments, such as subaerial hot springs (52, 53) and deep-sea hydrothermal vents (54); however, it is unclear whether any of these environments can also support the assembly of RNA, suggesting a possible unique role for soda lakes during the origin of life.

**Fig. 6.**
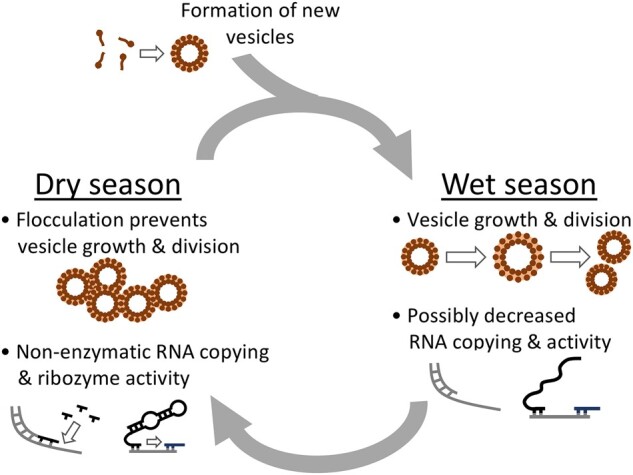
A hypothetical development cycle for protocells based on seasonal fluctuations in the water content of soda lakes.

In conclusion, we propose a seasonal model for protocell development in soda lakes on the early Earth (Fig. 6). We have shown that dry-season lake water containing ∼1 mM Mg^2+^ enables nonenzymatic RNA copying and ribozyme activity. Vesicle formation could occur during seasonally wet periods when the monovalent cation concentration is decreased by dilution, and those vesicles could retain encapsulated contents during transient flocculation in the dry season. Formation of vesicles de novo seems to be inefficient in dry-season soda lake water, and flocculation in dry-season water may prevent membrane growth and division. The formation of new vesicles, growth, and division of existing vesicles (55) could occur during the wet season, or in the dilute streams that feed into closed-basin lakes. Biopolymer building blocks, such as sugars, nucleobases (56), and amino acids (8), enable vesicles to resist flocculation in moderate concentrations of monovalent cations, which could prolong the seasonal duration of dispersed vesicles. Continued evaporation yields sodium concentrations above ∼1 M where vesicles flocculate despite the presence of additional compounds. Our findings are consistent with previous models for the origin of life that include a prominent role for seasonal evaporation (57). Taken together, our results suggest that natural soda lakes, which are especially common in regions of volcanic bedrock (26) that should dominate early Earth land surfaces (22), could have supported the formation of the earliest cellular life.

## Materials and methods

### Materials

RNAs and DNA were from Integrated DNA Technologies (Coralville, IA, USA). NaCl, Na_2_CO_3_, bis-tris-propane, formamide, decanol, and carboxyflorescein were from Sigma-Aldrich (St. Louis, MO, USA). One molar MgCl_2_ stock solution was from Ambion (Austin, TX, USA). Ultrapure water, 0.5 M ethylenediaminetetraacetic acid (EDTA), and SYBR Gold gel stain were from Invitrogen (Waltham, MA, USA). 220 nm nylon syringe filters were from RESTEK (Bellefonte, PA, USA). Decanoic acid was from Nu-Chek Prep (Elysian, MN, USA), and NaHCO_3_ was from EMD Chemicals (Darmstadt, Germany). Sepharose 4B was used for size-exclusion chromatography (Sigma-Aldrich).

### Sampling from Last Chance Lake and Goodenough Lake

Autoclaved glass bottles were filled with water from either Last Chance Lake or Goodenough Lake during November 2021 (27). The water was kept on ice for ∼6 hours during the return to the laboratory, at which point it was immediately frozen at −20 °C. Subsequently, the water was thawed and filtered to 220 nm to remove contaminants, such as sediment and microbes. The filtered water was again frozen at −20 or −30 °C. Aliquots of the lake water were kept refrigerated at 4 °C for up to a few weeks until used in experiments.

### Synthesis of 2-aminoimidazole-activated cytidine monophosphate nucleotides

As previously described (33), 1 equivalent of cytidine 5′-monophosphate (∼0.2 g) was mixed with 5.4 equivalents of 2-aminoimidazole hydrochloride and 8 equivalents of triphenylphosphine in dry dimethyl sulfoxide (4 mL) and triethylamine (1 mL). Then 7.3 equivalents of dipyridyl-disulfide were added. The mixture was incubated for 2 hours, then the product was precipitated in acetone (∼40 mL) with ∼2 mL of saturated sodium perchlorate in acetone. The precipitant was washed twice in 1:1 acetone:diethyl ether (v/v) and dried under vacuum. The dry pellet was resuspended in 0.1 M triethylamine bicarbonate at pH 8, and purified by reverse-phase flash chromatography, with a 50-g C18Aq column. The desired product was separated from other compounds over 12 column volumes of 0–10% acetonitrile in 2 mM triethylamine bicarbonate at pH 8. The purified product was immediately adjusted with NaOH so that pH was between 9.5 and 10, then the product was lyophilized and stored at −30 °C. Because of the adjustment to alkaline pH before lyophilization, 2-aminoimidazole-activated cytidine monophosphate nucleotides can be subsequently resuspended to high concentrations up to 400 mM.

### Nonenzymatic, RNA-templated extension of RNA primers

RNA primer-template duplexes were prepared by mixing primer and template stocks with lake water or artificial salt solutions. The RNA primer was labeled with 6-FAM at its 5′ end; the sequence of the primer was 5′-AGU GAG UAA CGG-3′. The sequence of the RNA template was 5′-GGGG CCG UUA CUC ACU AAA-3′. Cytidine 5′-monophosphate nucleotides activated by 2-aminoimidazole were resuspended in ultrapure water to 400 mM and kept on ice. A nucleotide solution was added to the primer-template duplex solution to initiate the reaction. All reactions were performed at room temperature. The final solution volume was 10 μL, and the solution contained 1 μM primer, 1.5 μM template, and 20 mM nucleotide. Reactions in lake water contained 90% natural water from either Last Chance Lake or Goodenough Lake. Reactions in laboratory standard conditions contained 50 mM MgCl_2_ and 0.2 M bis-tris-propane at pH 8, and reactions in artificial lake water contained 0.5 M Na_2_CO_3_, 1 mM MgCl_2_, and 0.2 M bis-tris-propane at pH 10. Aliquots of each reaction were quenched (50-fold dilution) at various times into a solution containing 4 μM of RNA that is complementary to the template (5′-UUU AGU GAG UAA CGG CCCC-3′) as well as 83.5% formamide (v), 25 mM EDTA, and 1× TBE (Tris/Borate/EDTA buffer, 0.1 M EDTA, 89 mM Tris, and 89 mM boric acid at pH 8.3). Primer extension products in the quenched reaction samples were resolved by 20% polyacrylamide gel electrophoresis in 1× TBE gel running buffer. The gels were scanned with an Amersham Typhoon RGB Biomolecular Imager (GE Healthcare Life Sciences). The fluorescently labeled primer and extended primer bands were visualized, and then quantified using ImageQuant TL software to obtain relative band intensities.

### Preparation of the ligase ribozyme

The ligase ribozyme *RS8 trunc* with sequence 5′-GGA CAG CGA GCC ACU GCG GAA GAC CUU AAG AGG UGU AAU UGC UCA CCC CGC UGU CCU UUU UUG GCU AAGG-3′ has been described previously (10, 45). The ribozyme was prepared by in vitro transcription of dsDNA templates with 2′-*O*-methyl modifications on the last two nucleotides (58). Transcription reactions were carried out in the presence of 40 mM Tris-HCl, 2 mM spermidine, 10 mM NaCl, 25 mM MgCl_2_, 10 mM dithiothreitol, 30 U/mL RNase inhibitor murine (New England Biolabs), 2.5 U/mL thermostable inorganic pyrophosphatase (TIPPase, New England Biolabs), 4 mM of each nucleotide triphosphate, 30 pmol/mL DNA template, and 1 U/µL T7 RNA Polymerase (New England Biolabs) at pH 8 for 3 hours at 37 °C. The reaction was quenched with DNase I (New England Biolabs), and RNA was extracted with phenol-chloroform-isoamyl alcohol, ethanol precipitated, and purified by denaturing polyacrylamide gel electrophoresis. The purified product was stored at −30 °C.

### Preparation of 2-aminoimidazole-activated RNA substrate for ligation

The 2-aminoimidazole-activated RNA substrate was generated from a 5′-monophosphorylated oligonucleotide (5′-ACC ACC GCA UUC CGCA-3′) by incubating it with 0.2 M 1-ethyl-3-(3-dimethylaminopropyl)carbodiimide (HCl salt) and 0.6 M 2-aminoimidazole (HCl salt, pH adjusted to 6) for 2 hours at room temperature. The product was washed five times with water in Amicon Ultra spin columns (3 kDa cutoff, 200 µL Milli-Q water per wash) and purified by reverse-phase analytical high-performance liquid chromatography using a gradient of 98 to 75% 20 mM triethylamine bicarbonate (pH 8) vs. acetonitrile over 40 min (45). The purified product was stored at −30 °C.

### Ribozyme-catalyzed ligation reactions

Ribozyme and template RNA (5′-GCG GUG GUC CUU AGCC-3′) were mixed with lake water or artificial salt solutions. The 2-aminoimidazole-activated substrate RNA was added to the ribozyme-template solution to initiate the reaction. All reactions were performed at room temperature. The final solution volume was 5 μL, and the solution contained 1 μM ribozyme, 1.2 μM template, and 2 μM substrate. Reactions contained 70% water from either Last Chance Lake or Goodenough Lake. For reactions with pH modified lake water, CO_2_ was bubbled through 1 mL of water from either Last Chance Lake or Goodenough Lake for ∼1 min. The modified lake water remained stable near pH 9 for at least 1 week, and aliquots of the same 1 mL modified lake water were used for all replicate experiments. Ribozyme reactions were quenched immediately and after 20 hours by adding 1 µL of reaction into 5 µL of 8 M urea, 0.1 M Tris-HCl, 0.1 M boric acid, and 0.1 M EDTA. Reaction products in the quenched samples were resolved by denaturing polyacrylamide gel electrophoresis in 1× TBE gel running buffer. Gels were stained using SYBR Gold then scanned with an Amersham Typhoon RGB Biomolecular Imager (GE Healthcare Life Sciences).

### Preparation of vesicles with natural soda lake water in the exterior solution

A stock solution of decanoate was made by dissolving solid decanoic acid in an equimolar NaOH solution, followed by gentle heating and rocking. Vesicle solutions were prepared at room temperature by the following steps: (i) Solutions of NaHCO_3_ and carboxyfluorescein dye were mixed. (ii) Decanoate solution was added. (iii) Liquid decanol was added and mixed to induce vesicle assembly. The resulting vesicles of 112.5 mM decanoic acid and 112.5 mM decanol encapsulated the bulk solution of 0.1 M NaHCO_3_ and 0.1 mM carboxyfluorescein. (iv) The pH was adjusted to 10 with NaOH after the amphiphiles were added. (v) The exterior solution around the vesicles was replaced with lake water (Fig. S13). By adding two volume equivalents of lake water to the initial vesicle sample, a layer of flocculated vesicles formed after gentle spinning for 10–30 s. The solution below this vesicle layer was removed. This replacement process was repeated five times. We are confident that this process produces vesicles that are surrounded by lake water because there is excellent contrast between encapsulated carboxyfluorescein and the exterior solution.

### Fluorescence microscopy

We used rhodamine 6G to stain vesicle membranes, and carboxyfluorescein to stain vesicle interiors. Samples were prepared for imaging by mixing 100 μL of vesicle solution with 1 μL of 1 mM rhodamine 6G stock. The edges of a cover slip were coated with vacuum grease, and 90 μL of sample was placed in the resulting well. Another cover slip was placed on top. Images were collected on a Nikon (Melville, NY, USA) Eclipse upright epifluorescence microscope (ME600L). A Chroma (Bellows Falls, VT, USA) FITC/Alexa488 filter cube was used for carboxyfluorescein fluorescence images, and a Chroma mCherry/Texas Red filter cube was used for rhodamine fluorescence images.

## Supplementary Material

pgae084_Supplementary_Data

## Data Availability

All data are included in the manuscript and supplementary material.

## Preprints

An earlier version of this manuscript was published as a chapter in Z.R.C.'s PhD dissertation (59).

## References

[1] Black RA, Blosser MC. 2016. A self-assembled aggregate composed of a fatty acid membrane and the building blocks of biological polymers provides a first step in the emergence of protocells. Life (Basel). 6:33. 27529283 10.3390/life6030033 PMC5041009

[2] Joyce GF, Szostak JW. 2018. Protocells and RNA self-replication. Cold Spring Harb Perspect Biol. 10:a034801. 30181195 10.1101/cshperspect.a034801 PMC6120706

[3] Mansy SS, et al 2008. Template-directed synthesis of a genetic polymer in a model protocell. Nature. 454:122–125. 18528332 10.1038/nature07018 PMC2743009

[4] Jin L, Engelhart AE, Zhang W, Adamala K, Szostak JW. 2018. Catalysis of template-directed nonenzymatic RNA copying by iron(II). J Am Chem Soc. 140:15016–15021. 30335371 10.1021/jacs.8b09617 PMC7547886

[5] Chen J, et al 2013. Identification of the catalytic Mg2+ ion in the hepatitis delta virus ribozyme. Biochemistry. 52:557–567. 23311293 10.1021/bi3013092 PMC3558840

[6] Monnard P-A, Apel CL, Kanavarioti A, Deamer DW. 2002. Influence of ionic inorganic solutes on self-assembly and polymerization processes related to early forms of life: implications for a prebiotic aqueous medium. Astrobiology. 2:139–152. 12469365 10.1089/15311070260192237

[7] Adamala K, Szostak JW. 2013. Nonenzymatic template-directed RNA synthesis inside model protocells. Science. 342:1098–1100. 24288333 10.1126/science.1241888 PMC4104020

[8] Cornell CE, et al 2019. Prebiotic amino acids bind to and stabilize prebiotic fatty acid membranes. Proc Natl Acad Sci U S A. 116:17239–17244. 31405964 10.1073/pnas.1900275116 PMC6717294

[9] Chen IA, Salehi-Ashtiani K, Szostak JW. 2005. RNA catalysis in model protocell vesicles. J Am Chem Soc. 127:13213–13219. 16173749 10.1021/ja051784p PMC5072289

[10] DasGupta S, Zhang SJ, Smela MP, Szostak JW. 2023. RNA-catalyzed RNA ligation within prebiotically plausible model protocells. Chemistry. 29:e202301376. 10.1002/chem.202301376 37216492

[11] Boros E, Kolpakova M. 2018. A review of the defining chemical properties of soda lakes and pans: an assessment on a large geographic scale of Eurasian inland saline surface waters. PLoS One. 13:e0202205. 30125301 10.1371/journal.pone.0202205 PMC6101393

[12] Zorz JK, et al 2019. A shared core microbiome in soda lakes separated by large distances. Nat Commun. 10:4230. 31530813 10.1038/s41467-019-12195-5 PMC6748926

[13] Toner JD, Catling DC. 2020. A carbonate-rich lake solution to the phosphate problem of the origin of life. Proc Natl Acad Sci U S A. 117:883–888. 31888981 10.1073/pnas.1916109117 PMC6969521

[14] Toner JD, Catling DC. 2019. Alkaline lake settings for concentrated prebiotic cyanide and the origin of life. Geochim Cosmochim Acta. 260:124–132.

[15] Ranjan S, Todd ZR, Sutherland JD, Sasselov DD. 2018. Sulfidic anion concentrations on early Earth for surficial origins-of-life chemistry. Astrobiology. 18:1023–1040. 29627997 10.1089/ast.2017.1770 PMC6225604

[16] Patel BH, Percivalle C, Ritson DJ, Duffy CD, Sutherland JD. 2015. Common origins of RNA, protein and lipid precursors in a cyanosulfidic protometabolism. Nat Chem. 7:301–307. 25803468 10.1038/nchem.2202 PMC4568310

[17] Xu J, et al 2018. Photochemical reductive homologation of hydrogen cyanide using sulfite and ferrocyanide. Chem Commun (Camb). 54:5566–5569. 29761807 10.1039/c8cc01499j PMC5972737

[18] Lawless JG, Yuen GU. 1979. Quantification of monocarboxylic acids in the murchison carbonaceous meteorite. Nature. 282:396–398.

[19] Nooner DW, Oro J. 1979. Synthesis of fatty acids by a closed system Fischer-Tropsch process. In: Hydrocarbon synthesis from carbon monoxide and hydrogen, Advances in Chemistry. American Chemical Society. p. 159–171.

[20] Criado-Reyes J, Bizzarri BM, García-Ruiz JM, Saladino R, Di Mauro E. 2021. The role of borosilicate glass in Miller–Urey experiment. Sci Rep. 11:21009. 34697338 10.1038/s41598-021-00235-4 PMC8545935

[21] Cohen ZR, et al 2023. Plausible sources of membrane-forming fatty acids on the early Earth: a review of the literature and an estimation of amounts. ACS Earth Space Chem. 7:11–27. 36704178 10.1021/acsearthspacechem.2c00168 PMC9869395

[22] Korenaga J . 2021. Was there land on the early Earth? Life (Basel). 11:1142. 34833018 10.3390/life11111142 PMC8623345

[23] Harrison TM, Bell EA, Boehnke P. 2017. Hadean zircon petrochronology. Rev Mineral Geochem. 83:329–363.

[24] Rosas JC, Korenaga J. 2021. Archaean seafloors shallowed with age due to radiogenic heating in the mantle. Nat Geosci. 14:51–56.

[25] Aguirre-Garrido JF, Ramírez-Saad HC, Toro N, Martínez-Abarca F. 2016. Bacterial diversity in the soda saline crater lake from Isabel Island, Mexico. Microb Ecol. 71:68–77. 26391805 10.1007/s00248-015-0676-6

[26] Schagerl M, Renaut RW. 2016. Dipping into the soda lakes of East Africa. In: Schagerl M, editor. Soda lakes of East Africa. Cham: Springer International Publishing. p. 3–24.

[27] Haas S, Sinclair KP, Catling DC. 2024. Biogeochemical explanations for the world's most phosphate-rich lake, an origin-of-life analog. Commun Earth Environ. 5:1–11.

[28] Rajamani S, et al 2008. Lipid-assisted synthesis of RNA-like polymers from mononucleotides. Orig Life Evol Biosph. 38:57–74. 18008180 10.1007/s11084-007-9113-2

[29] Kanavarioti A, Monnard P-A, Deamer DW. 2001. Eutectic phases in ice facilitate nonenzymatic nucleic acid synthesis. Astrobiology. 1:271–281. 12448990 10.1089/15311070152757465

[30] Ferris JP, Ertem G. 1992. Oligomerization of ribonucleotides on montmorillonite: reaction of the 5′-phosphorimidazolide of adenosine. Science. 257:1387–1389. 1529338 10.1126/science.1529338

[31] Walton T, Szostak JW. 2016. A highly reactive imidazolium-bridged dinucleotide intermediate in nonenzymatic RNA primer extension. J Am Chem Soc. 138:11996–12002. 27552367 10.1021/jacs.6b07977 PMC6326528

[32] Duzdevich D, et al 2021. Competition between bridged dinucleotides and activated mononucleotides determines the error frequency of nonenzymatic RNA primer extension. Nucleic Acids Res. 49:3681–3691. 33744957 10.1093/nar/gkab173 PMC8053118

[33] Ding D, Zhou L, Giurgiu C, Szostak JW. 2022. Kinetic explanations for the sequence biases observed in the nonenzymatic copying of RNA templates. Nucleic Acids Res. 50:35–45. 34893864 10.1093/nar/gkab1202 PMC8754633

[34] Zhang SJ, Duzdevich D, Ding D, Szostak JW. 2022. Freeze-thaw cycles enable a prebiotically plausible and continuous pathway from nucleotide activation to nonenzymatic RNA copying. Proc Natl Acad Sci U S A. 119:e2116429119. 10.1073/pnas.2116429119 PMC9169909 35446612

[35] Giurgiu C, et al 2021. Structure–activity relationships in nonenzymatic template-directed RNA synthesis. Angew Chem Int Ed Engl. 60:22925–22932. 34428345 10.1002/anie.202109714 PMC8490286

[36] Li Y, Breaker RR. 1999. Kinetics of RNA degradation by specific base catalysis of transesterification involving the 2′-hydroxyl group. J Am Chem Soc. 121:5364–5372.

[37] Guth-Metzler R, et al 2023. Goldilocks and RNA: where Mg2+ concentration is just right. Nucleic Acids Res. 51:3529–3539. 36987860 10.1093/nar/gkad124 PMC10164553

[38] Aitken HRM, Wright TH, Radakovic A, Szostak JW. 2023. Small-molecule organocatalysis facilitates in situ nucleotide activation and RNA copying. J Am Chem Soc. 145:16142–16149. 37431761 10.1021/jacs.3c04635 PMC10375519

[39] Prywes N, Blain JC, Del Frate F, Szostak JW. 2016. Nonenzymatic copying of RNA templates containing all four letters is catalyzed by activated oligonucleotides. Elife. 5:e17756. 27351102 10.7554/eLife.17756 PMC4959843

[40] Ding D, Zhang SJ, Szostak JW. 2023. Enhanced nonenzymatic RNA copying with in-situ activation of short oligonucleotides. Nucleic Acids Res. 51:6528–6539. 37247941 10.1093/nar/gkad439 PMC10359593

[41] Wu T, Orgel LE. 1992. Nonenzymatic template-directed synthesis on hairpin oligonucleotides. 2. Templates containing cytidine and guanosine residues. J Am Chem Soc. 114:5496–5501. 11538402 10.1021/ja00040a002

[42] Zhou L, Ding D, Szostak JW. 2021. The virtual circular genome model for primordial RNA replication. RNA. 27:1–11. 33028653 10.1261/rna.077693.120 PMC7749632

[43] Ding D, Zhou L, Mittal S, Szostak JW. 2023. Experimental tests of the virtual circular genome model for nonenzymatic RNA replication. J Am Chem Soc. 145:7504–7515. 36963403 10.1021/jacs.3c00612 PMC10080680

[44] Kadoya S, Krissansen-Totton J, Catling DC. 2020. Probable cold and alkaline surface environment of the hadean Earth caused by impact ejecta weathering. Geochem Geophys Geosyst. 21:e2019GC008734.

[45] Walton T, DasGupta S, Duzdevich D, Oh SS, Szostak JW. 2020. In vitro selection of ribozyme ligases that use prebiotically plausible 2-aminoimidazole–activated substrates. Proc Natl Acad Sci U S A. 117:5741–5748. 32123094 10.1073/pnas.1914367117 PMC7084097

[46] Cohen ZR, Cornell CE, Catling DC, Black RA, Keller SL. 2022. Prebiotic protocell membranes retain encapsulated contents during flocculation, and phospholipids preserve encapsulation during dehydration. Langmuir. 38:1304–1310. 35026114 10.1021/acs.langmuir.1c03296

[47] Fahrenbach AC, et al 2017. Common and potentially prebiotic origin for precursors of nucleotide synthesis and activation. J Am Chem Soc. 139:8780–8783. 28640999 10.1021/jacs.7b01562 PMC6326526

[48] Szostak JW . 2012. The eightfold path to non-enzymatic RNA replication. J Syst Chem. 3:2.

[49] Kühne H, Joyce GF. 2003. Continuous in vitro evolution of ribozymes that operate under conditions of extreme pH. J Mol Evol. 57:292–298. 14629039 10.1007/s00239-003-2480-z

[50] O’Flaherty DK, et al 2018. Copying of mixed-sequence RNA templates inside model protocells. J Am Chem Soc. 140:5171–5178. 29608310 10.1021/jacs.8b00639 PMC7547884

[51] Khvorova A, Kwak Y-G, Tamkun M, Majerfeld I, Yarus M. 1999. RNAs that bind and change the permeability of phospholipid membranes. Proc Natl Acad Sci U S A. 96:10649–10654. 10485880 10.1073/pnas.96.19.10649 PMC17937

[52] Joshi MP, Samanta A, Tripathy GR, Rajamani S. 2017. Formation and stability of prebiotically relevant vesicular systems in terrestrial geothermal environments. Life (Basel). 7:51. 29189763 10.3390/life7040051 PMC5745564

[53] Milshteyn D, Damer B, Havig J, Deamer D. 2018. Amphiphilic compounds assemble into membranous vesicles in hydrothermal hot spring water but not in seawater. Life (Basel). 8:11. 29748464 10.3390/life8020011 PMC6027054

[54] Jordan SF, et al 2019. Promotion of protocell self-assembly from mixed amphiphiles at the origin of life. Nat Ecol Evol. 3:1705–1714. 31686020 10.1038/s41559-019-1015-y

[55] Zhu TF, Szostak JW. 2009. Coupled growth and division of model protocell membranes. J Am Chem Soc. 131:5705–5713. 19323552 10.1021/ja900919c PMC2669828

[56] Black RA, et al 2013. Nucleobases bind to and stabilize aggregates of a prebiotic amphiphile, providing a viable mechanism for the emergence of protocells. Proc Natl Acad Sci U S A. 110:13272–13276. 23901105 10.1073/pnas.1300963110 PMC3746888

[57] Damer B, Deamer D. 2019. The hot spring hypothesis for an origin of life. Astrobiology. 20:429–452. 31841362 10.1089/ast.2019.2045 PMC7133448

[58] Kao C, Rüdisser S, Zheng M. 2001. A simple and efficient method to transcribe RNAs with reduced 3′ heterogeneity. Methods. 23:201–205. 11243833 10.1006/meth.2000.1131

[59] Cohen ZR . 2023. Testing hypotheses for the formation of the first cells within plausible early Earth environments. University of Washington. Doctoral dissertation.

