# Supplementary material for: Natural soda lakes provide compatible conditions for RNA and membrane function that could have enabled the origin of life
Source: PNAS Nexus. 2024 Mar 19;3(3):pgae084. doi: 10.1093/pnasnexus/pgae084 (PMC10949909; doi:10.1093/pnasnexus/pgae084)
Supplement: pgae084_Supplementary_Data [file pgae084_supplementary_data.pdf]

# Supporting Information for

## Natural soda lakes provide compatible conditions for RNA and membrane function that could have enabled the origin of life

Zachary R. Cohen*, Dian Ding, Lijun Zhou, Saurja DasGupta, Sebastian Haas, Kimberly Sinclair, Zoe R. Todd, Roy A. Black, Jack W. Szostak*, David C. Catling*

*co-corresponding authors: Zachary R. Cohen, Jack W. Szostak, David C. Catling
Email: zrcohen2@uw.edu, jwszostak@uchicago.edu, dcatling@uw.edu

**This PDF file includes:**

Figures S1 to S17
Tables S1 to S3
SI References

**Table of Contents:**

Section 1: Ionic composition of Last Chance Lake and Goodenough Lake

Section 2: Non-enzymatic, RNA-templated extension of RNA primers by 2-aminoimidazole activated ribonucleotides

Section 3: Activity of a ligase ribozyme

Section 4: Encapsulation by prebiotic membranes

Section 5: SI references

**Section 1: Ionic composition of Last Chance Lake and Goodenough Lake**

**Table S1.** Cation composition of Last Chance Lake and Goodenough Lake measured by inductively coupled plasma mass spectrometry

| Ion name | Concentration in Last Chance Lake (mM) | Concentration in Goodenough Lake (mM) |
|---|---|---|
| Sodium | 1824 ± 4 | 711.4 ± 4.0 |
| Potassium | 28.3 ± 0.1 | 13.2 ± 0.1 |
| Monovalent cation total | 1852 | 724.6 |
| | | |
| Magnesium | 1.49 ± 0.03 | 1.50 ± 0.03 |
| Calcium | 0.094 ± 0.084 | < 0.01 |
| Divalent cation total | 1.58 | 1.50 |

**Table S2.** Anion composition of Last Chance Lake and Goodenough Lake

| Ion name | Concentration in Last Chance Lake (mM) | Concentration in Goodenough Lake (mM) | Analytical technique used |
|---|---|---|---|
| Chloride | 336 ± 1 | 70.4 ± 0.8 | Ion chromatography |
| Nitrate | < 0.001 | < 0.001 | Spectrophotometry (1) |
| Phosphate | 8.2 ± 0.1 | < 0.1 | Spectrophotometry (2) |
| Sulfate | 49.5 ± 0.2 | 7.6 ± 0.2 | Ion chromatography |
| Carbonate & bicarbonate | 899 ± 11 | 156 ± 6 | Shimadzu total organic carbon/total nitrogen-VCSH |

**Table S3.** pH of Last Chance Lake and Goodenough Lake

| | Last Chance Lake | Goodenough Lake |
|---|---|---|
| pH | 9.8 | 10.3 |

Concentration data are reproduced from Haas et al. 2024 with permission from the authors (3). All data are single measurements of water samples collected during November 2021. Uncertainties indicate measurement precision at the applied level of dilution.

**Section 2: Non-enzymatic, RNA-templated extension of RNA primers by 2-aminoimidazole activated ribonucleotides**

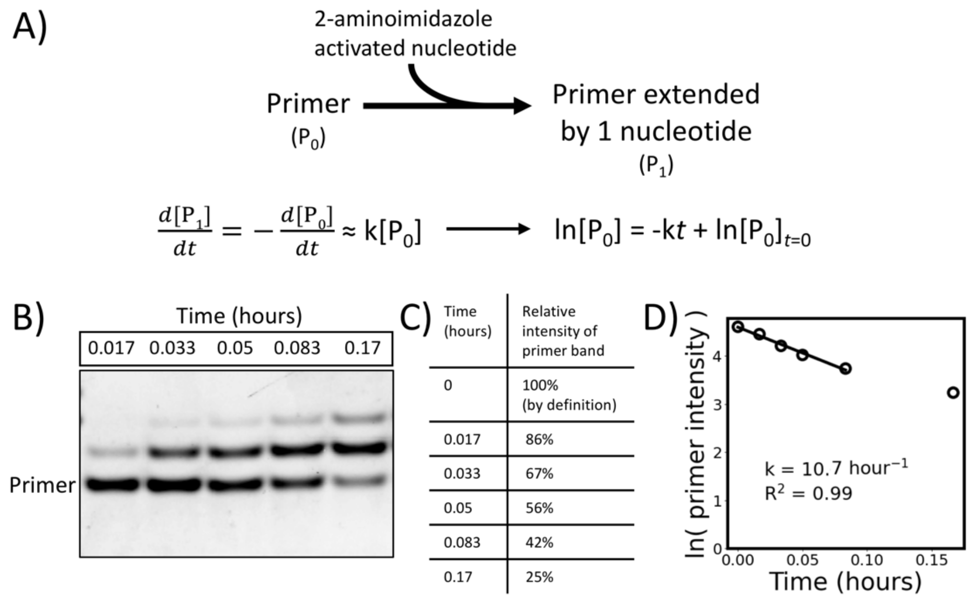


**Fig. S1.** Example calculation of the pseudo first-order rate constant for primer extension by the first nucleotide. A) When the concentration of 2-aminoimidazole activated nucleotides (20 mM) is in excess of the primer concentration (1 μM), the rate of production of primers that have been extended by 1 nucleotide is equal to the rate of consumption of unextended primer, and these rates depend approximately linearly on the concentration of unextended primer. The first-order integrated rate law can be used to estimate the rate constant, k. B) Example gel with labeled bands corresponding to the unextended primer. Bands corresponding to extended primers can be seen as well. These data are from 1 replicate experiment in artificial lake conditions from Fig. 2. C) Relative intensities of the unextended primer band can be quantified from the gel by fluorescence. By definition, none of the primer is extended at 0 hours. D) The negative slope of a plot of the natural logarithm of the unextended primer intensity vs time yields the pseudo first-order rate constant for primer extension by the first nucleotide.

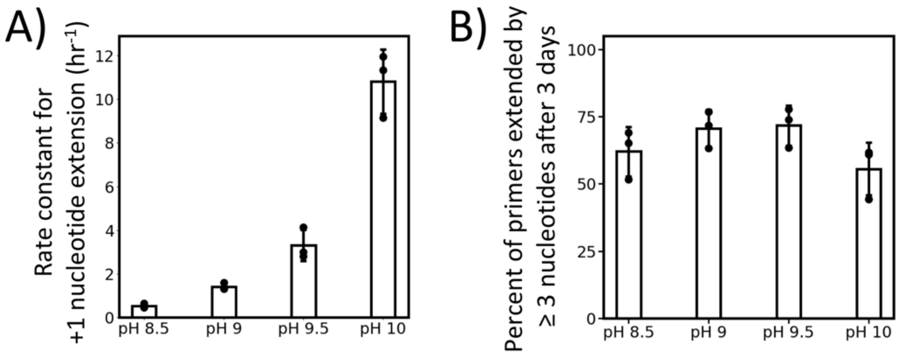


**Fig. S2.** In artificial lake water, A) pseudo first-order rate constants for primer extension increase with pH while B) the yield of extended primers after 3 days is largely unaffected by pH. All reactions contained 1 μM primer, 1.5 μM template, 20 mM nucleotide, 1 M NaCl, 1 mM $MgCl_2$, and 200 mM bis-tris-propane. Error bars correspond to the standard deviation from 3 independent experiments.

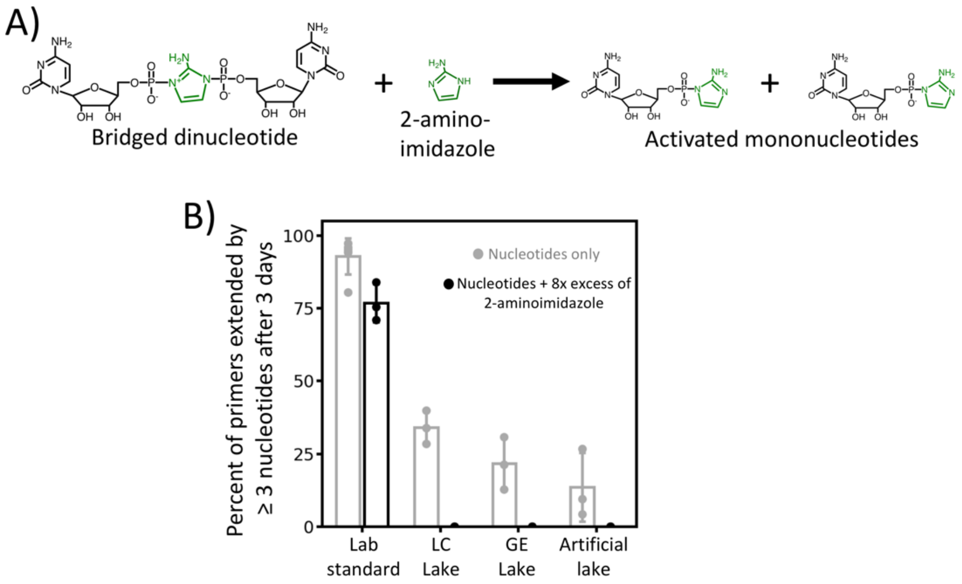


**Fig. S3.** Excess 2-aminoimidazole disrupts primer extension in lake water, suggesting that primer extension in lake water occurs via bridged dinucleotide intermediates. A) Excess 2-aminoimidazole disfavors formation of bridged dinucleotides. B) Comparison of primer extension in the presence or absence of excess 2-aminoimidazole. Data for “nucleotides only” are the same as in Fig. 3, (20 mM nucleotide). Reactions with 8x excess of 2-aminoimidazole were performed similarly as described for “nucleotides only”, except that 400 mM stock solutions of activated cytidine monophosphate ribonucleotides were resuspended in 3.2 M 2-aminoimidazole solution at pH 7, rather than ultrapure water. The final solution composition was 20 mM nucleotide, 160 mM 2-aminoimidazole, 1 μM primer, and 1.5 μM template. Standard lab conditions are 50 mM $MgCl_2$ and 200 mM bis-tris-propane at pH 8, and artificial lake water is 0.5 M $Na_2CO_3$, 1 mM $MgCl_2$, and 200 mM bis-tris-propane at pH 10. Error bars correspond to the standard deviation from 3 independent experiments, while the error bars in “nucleotides only” in lab standard conditions correspond to 6 independent experiments. LC is Last Chance Lake and GE is Goodenough Lake.

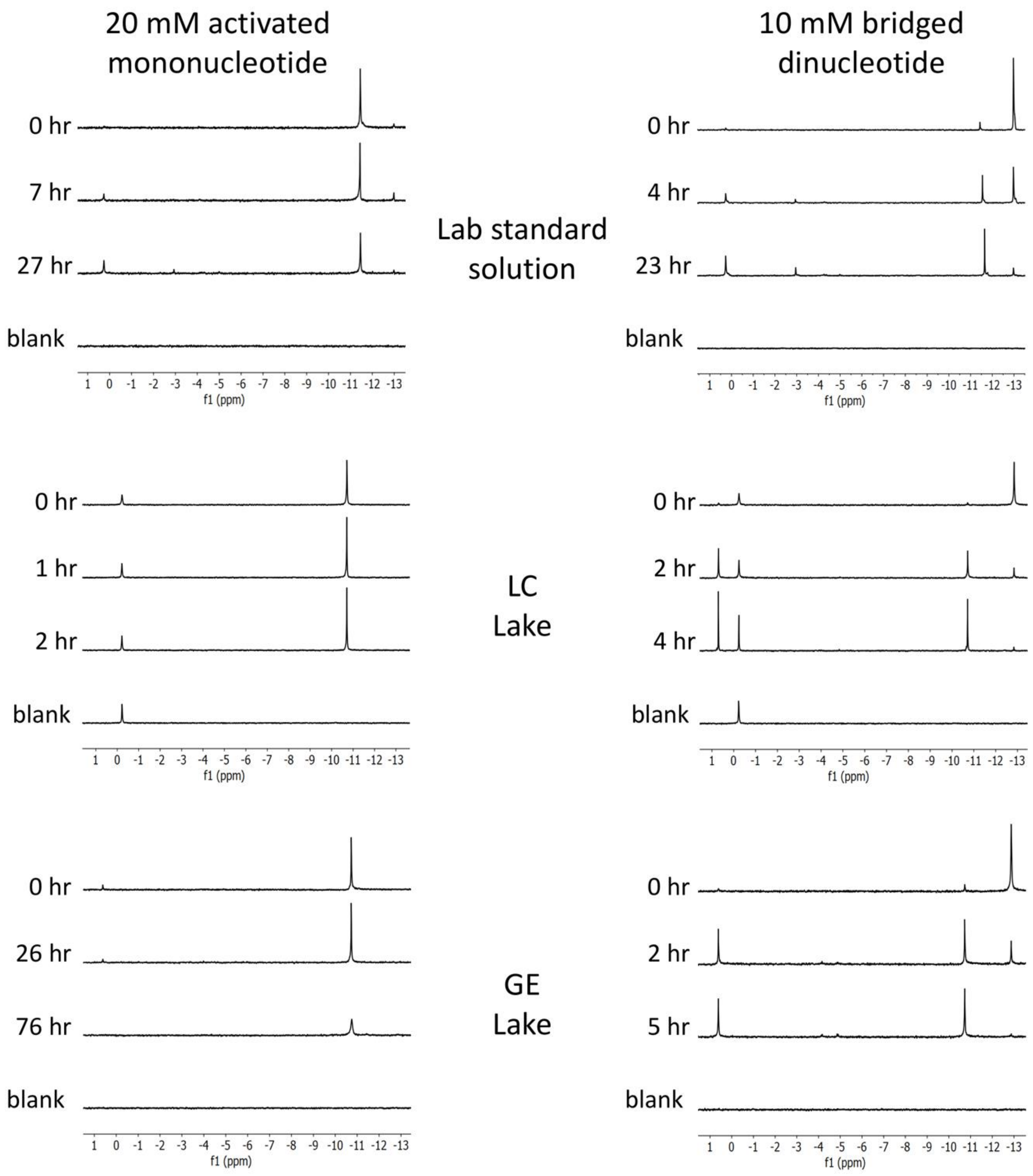


**Fig. S4.** $^{31}P$ NMR spectra of 2-aminoimidazole activated cytidine mononucleotide (-11.38 ppm) or bridged cytidine dinucleotide (-12.85 ppm). In standard lab conditions (50 mM $MgCl_2$ and 200 mM bis-tris-propane at pH 8), the bridged dinucleotide hydrolyzes slowly until it reaches a low equilibrium concentration after ~24 hours. In natural water from either Last Chance Lake (LC) or Goodenough Lake (GE), the activated mononucleotide is relatively stable throughout the experiment, whereas the bridged dinucleotide hydrolyzes rapidly within ~ 5 hours. For all samples, 10% $D_2O$ was added to the solution before dissolving the solid mononucleotide or dinucleotide. Additional signals correspond to phosphate (-0.3 ppm) and cytidine monophosphate (0.28 ppm). $^{31}P$ spectra were acquired on a Varian Oxford AS-400 NMR spectrometer. Bridged cytidine dimers were prepared as described in Ding et al. 2022 (4).

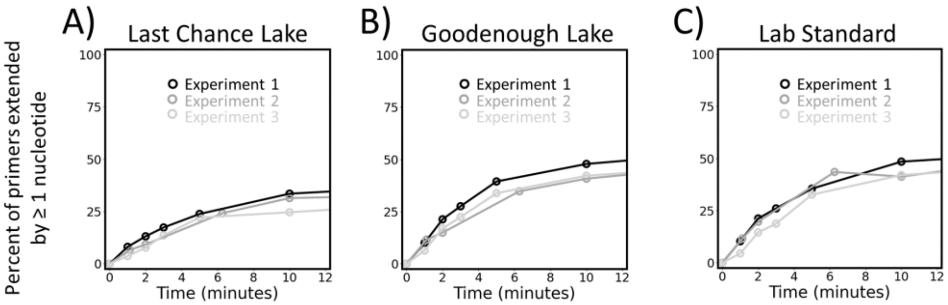


**Fig. S5.** In natural lake samples and standard laboratory conditions, there is an initial ~5 min period where the rate of primer extension is maximum, followed by sustained extension at a lower rate. All reactions contained 1 μM primer, 1.5 μM template, and 20 mM nucleotide. Standard lab conditions are 50 mM $MgCl_2$ and 200 mM bis-tris-propane at pH 8.

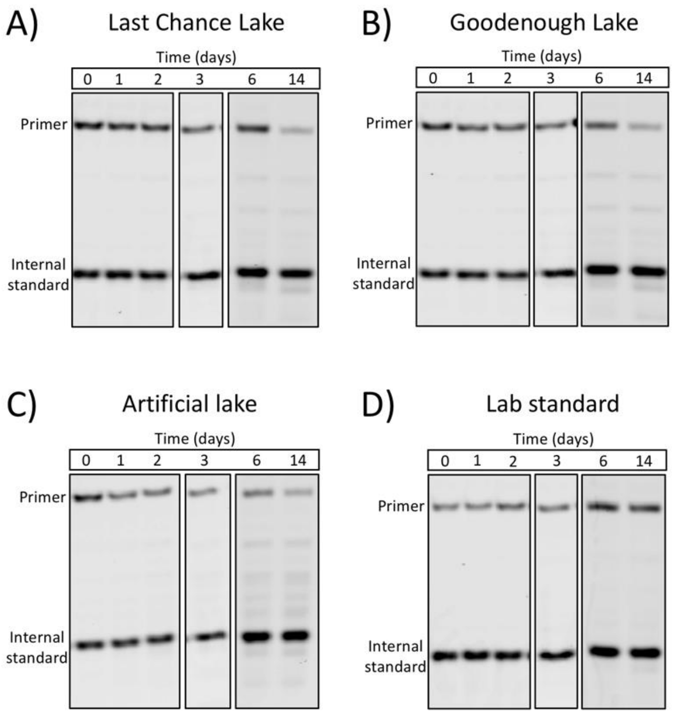


**Fig. S6.** In the absence of template RNA or activated nucleotides, the RNA primer is stable in lake water for at least 3 days. However, the primer begins to hydrolyze after 6 days in lake water. The primer is stable for 14 days in standard lab conditions. Standard lab conditions are 50 mM $MgCl_2$ and 200 mM bis-tris-propane at pH 8, and artificial lake water is 1 M NaCl, 1 mM $MgCl_2$, and 200 mM bis-tris-propane at pH 10.

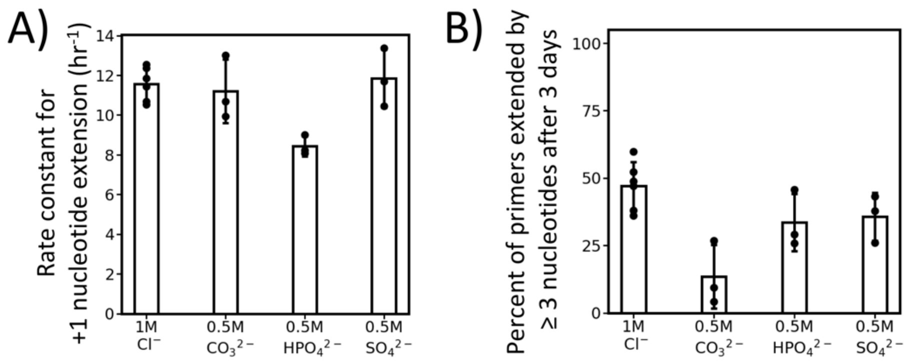


**Fig. S7.** In artificial lake water containing 1 M $Na^{+}$ and 1 mM $Mg^{2+}$ at pH 10, A) the pseudo first-order rate constants for primer extension are similar regardless of whether chloride, carbonate, phosphate, or sulfate are used as anions. Data for "0.5 M $CO_3^{2-}$" are the same as in Fig. 2C "Artificial Lake". B) The yield of extended primers after 3 days is lower when carbonate is used as the anion. Data for "0.5 M $CO_3^{2-}$" are the same as in Fig. 3 "Artificial Lake, 20 mM initial nucleotide". All reactions contained 1 μM primer, 1.5 μM template, 20 mM nucleotide, and 200 mM bis-tris-propane. Error bars correspond to the standard deviation from 3 independent experiments, except for the error bars in the "1 M $Cl^{-}$" sample which correspond to 6 independent experiments.

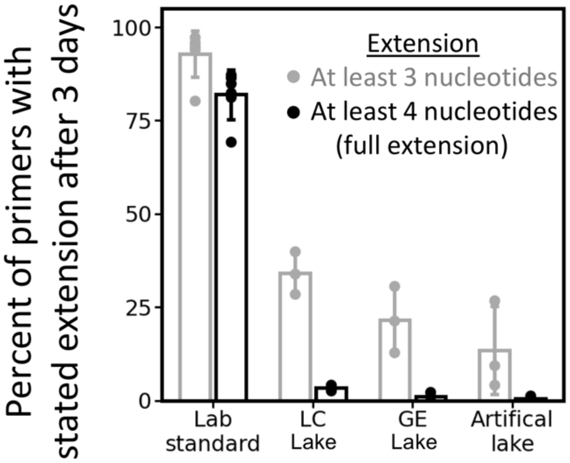


**Fig. S8.** The yield of fully extended RNA primers (+4 nucleotides) is low in soda lake water. All reactions contained 1 μM primer, 1.5 μM template, and 20 mM nucleotide. Standard lab conditions are 50 mM $MgCl_2$ and 200 mM bis-tris-propane at pH 8, and artificial lake water is 0.5 M $Na_2CO_3$, 1 mM $MgCl_2$, and 200 mM bis-tris-propane at pH 10. Error bars correspond to the standard deviation from 3 independent experiments, except for the error bars for the lab standard conditions which correspond to 6 independent experiments. LC is Last Chance Lake and GE is Goodenough Lake. Data for extension by at least 3 nucleotides are the same as in Fig. 3 "20 mM initial nucleotide".

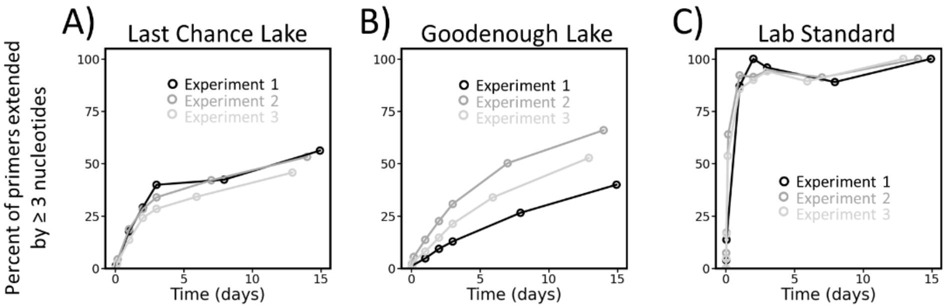


**Fig. S9.** The yield of RNA primers that have been extended by +3 nucleotides increases beyond 3 days. All reactions contained 1 μM primer, 1.5 μM template, and 20 mM nucleotide. Standard lab conditions are 50 mM $MgCl_2$ and 200 mM bis-tris-propane at pH 8.

**Section 3: Activity of a ligase ribozyme**

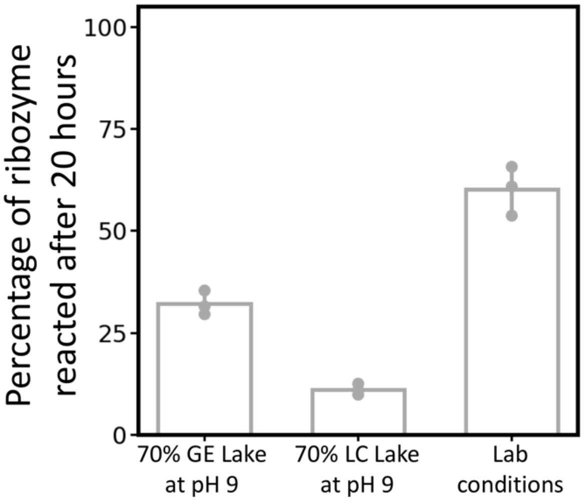


**Fig. S10.** The yield of reacted ribozyme after 20 hours in pH modified water from Goodenough Lake (GE) or Last Chance Lake (LC). Lab conditions are 0.362 M NaCl, 1.2 mM $MgCl_2$, and 242 mM bis-tris-propane at pH 8. The final solution volume was 5 μL, and the solution contained 1 μM ribozyme, 1.2 μM template, and 2 μM substrate. The error bars correspond to standard deviation from three independent experiments.

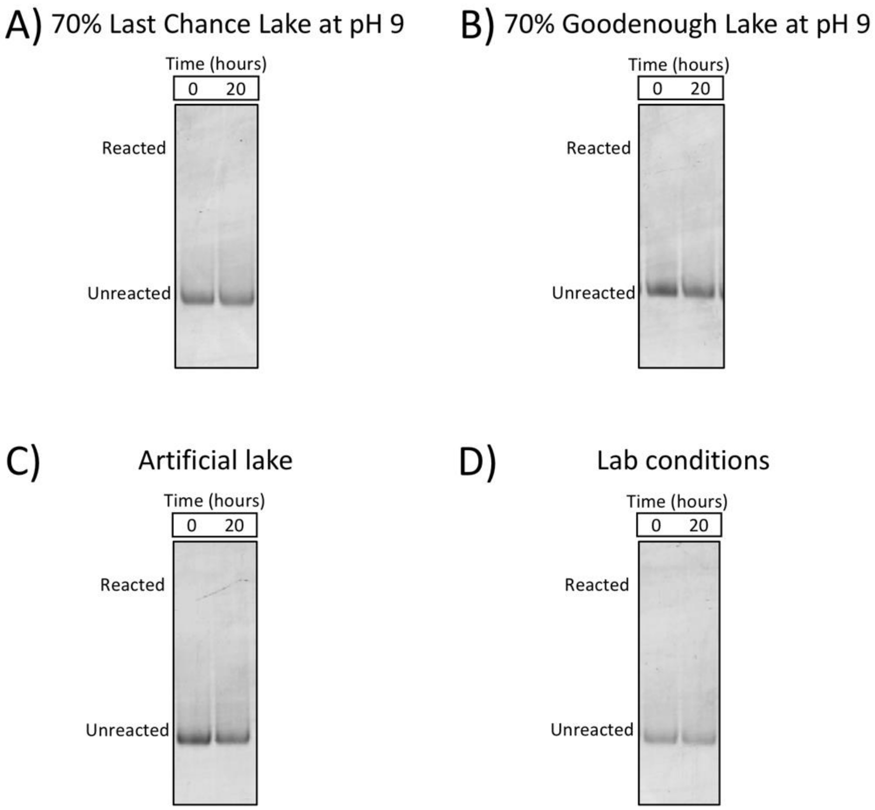


**Fig. S11.** In control reactions using an RNA construct with a completely randomized catalytic domain sequence instead of the ligase ribozyme sequence, we do not observe ligation to the substrate. The reagent composition was otherwise identical to the ribozyme reactions described in the main text: the solution contained 1.2 μM template, 2 μM substrate, and 1 μM of the 95 nucleotide RNA. Artificial lake conditions are 0.5 M $Na_2CO_3$, 1 mM $MgCl_2$, and 200 mM bis-tris-propane at pH 9, and lab conditions are 0.362 M NaCl, 1.2 mM $MgCl_2$, and 242 mM bis-tris-propane at pH 8. The data for each solution are from a single experiment, and the results are consistent with two additional, independent replicate experiments.

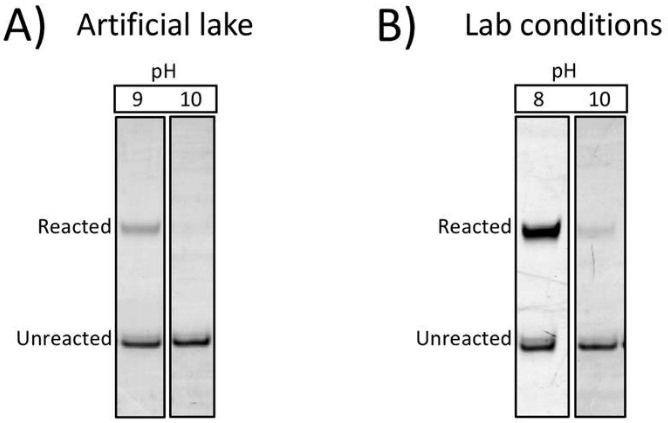


**Fig. S12.** Whether or not the salt composition mimics the natural lakes, ribozyme activity is higher when the pH is lowered below pH 10. A) In artificial lake conditions (0.5 M $Na_2CO_3$, 1 mM $MgCl_2$, and 200 mM bis-tris-propane), more ribozyme has reacted after 20 hours in pH 9 solution than pH 10 solution. B) In lab conditions (0.362 M NaCl, 1.2 mM $MgCl_2$, and 242 mM bis-tris-propane), more ribozyme has reacted after 20 hours in pH 8 solution than pH 10 solution. The final solution volume was 5 μL, and the solution contained 1 μM ribozyme, 1.2 μM template, and 2 μM substrate.

**Section 4: Encapsulation by prebiotic membranes**

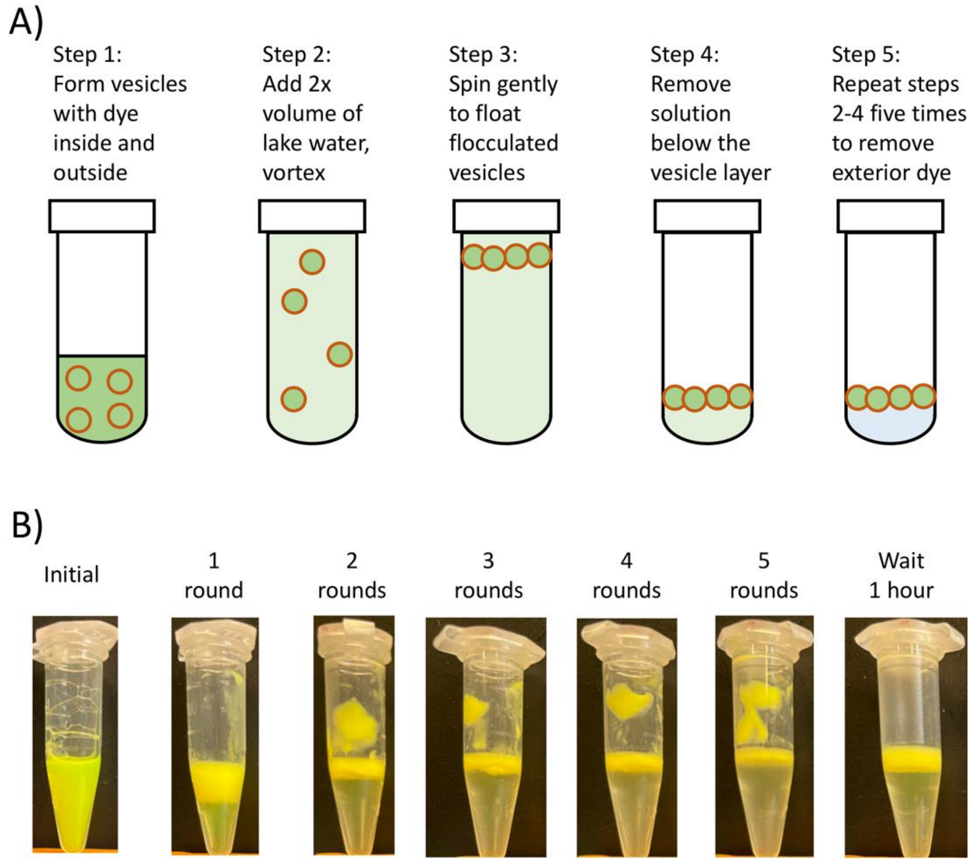


**Fig. S13.** A) Schematic of the process used to exchange the exterior solution around vesicles with lake water. B) Example of this process used to replace the exterior solution of vesicles with water from Last Chance Lake. The initial vesicle sample is 112.5 mM decanoic acid, 112.5 mM decanol, 0.1 M $NaHCO_3$, and 0.1 mM carboxyfluorescein. After 5 rounds of solution replacement, the resulting layer of flocculated vesicles is stable for at least 1 hour.

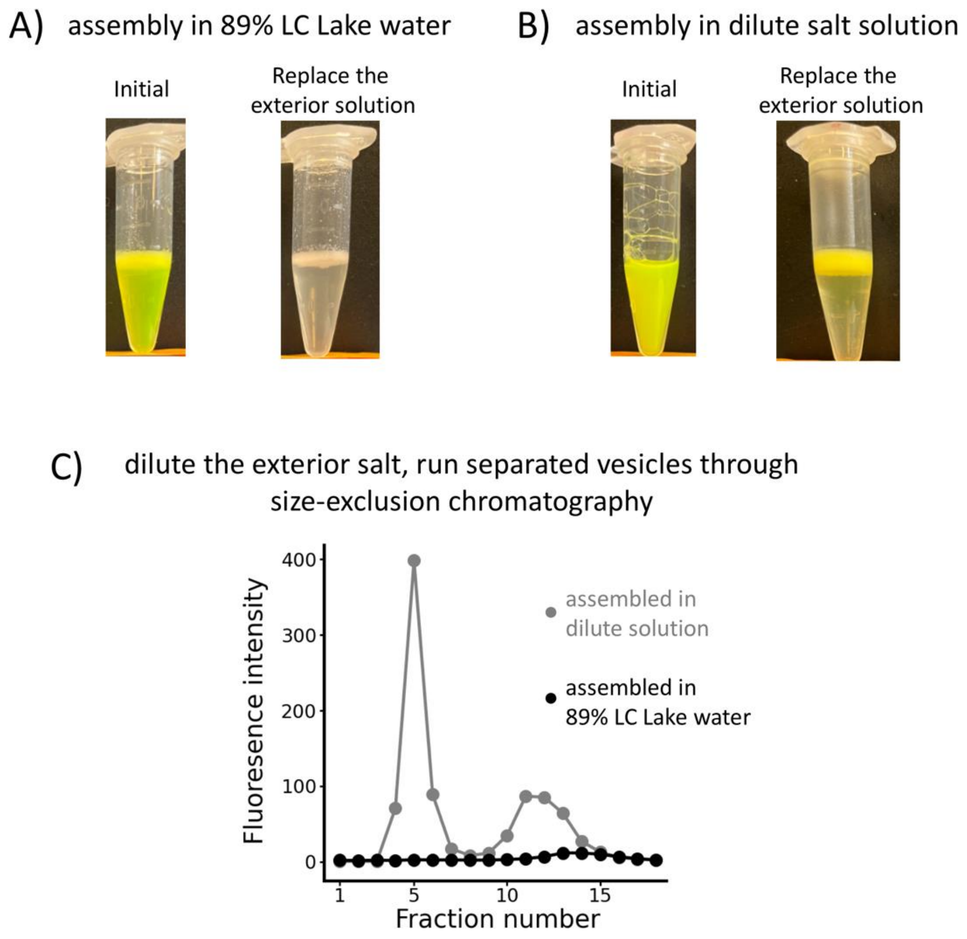


**Fig. S14.** Prebiotic amphiphiles do not assemble into vesicles in 89% water from the dry season of Last Chance Lake (LC). A) When 112.5 mM decanoic acid and 112.5 mM decanol were allowed to assemble in 89% Last Chance Lake water containing 0.1 mM carboxyfluorescein, the amphiphiles immediately formed a floating layer. The solution below this layer was replaced with 100% Last Chance Lake water as in Fig. S13. The amphiphile layer did not retain carboxyfluorescein. B) As described in Fig. 5 of the main text, 112.5 mM decanoic acid and 112.5 mM decanol were allowed to assemble into vesicles in a dilute salt solution of 0.1 M $NaHCO_3$ and 0.1 mM carboxyfluorescein. After replacing the exterior solution with 100% Last Chance Lake water as in Fig. S13, the vesicle layer did retain encapsulated carboxyfluorescein. C) After 1 hour, both samples were subsequently diluted into a 56 mM decanoic acid and 0.1 M $NaHCO_3$, pH 10 solution. If amphiphiles from the 89% Last Chance Lake sample assembled into vesicles following this dilution, those vesicles would not encapsulate dye. These diluted samples were eluted through size-exclusion columns. For the sample where amphiphiles were initially allowed to assemble in 89% Last Chance Lake water, the chromatogram does not show an early-eluting peak, suggesting that very few vesicles contain carboxyfluorescein. Meanwhile, there is an intense early-eluting peak due to vesicles containing carboxyfluorescein from the sample that was in dilute solution. These data suggest that amphiphiles did not assemble into new vesicles in Last Chance Lake water. Carboxyfluorescein fluorescence was measured with excitation at 485 nm and emission at 520 nm on a Thermo Labsystems Fluoroskan Ascent FL Fluorescence Microplate Reader. Data are from a single experiment, and they are consistent with an independent, replicate experiment.

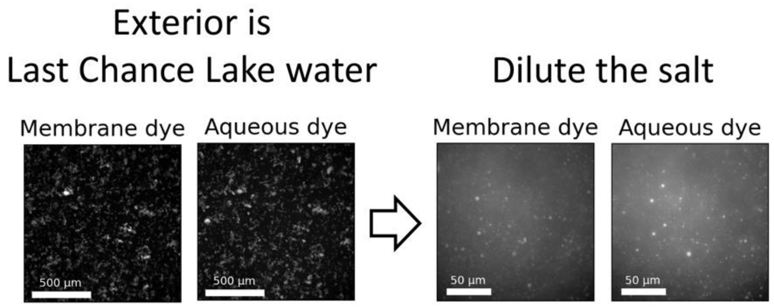


**Fig. S15.** Images of a field of vesicles composed of 1:1 decanoic acid: decanol when the exterior solution is water from Last Chance Lake, and after subsequent dilution into a 56 mM decanoic acid and 0.1 M $NaHCO_3$, pH 10 solution. Rhodamine 6G membrane dye was added immediately prior to imaging. Although these images are not the same field shown in Fig. 5 of the main text, they are also representative of the entire sample.

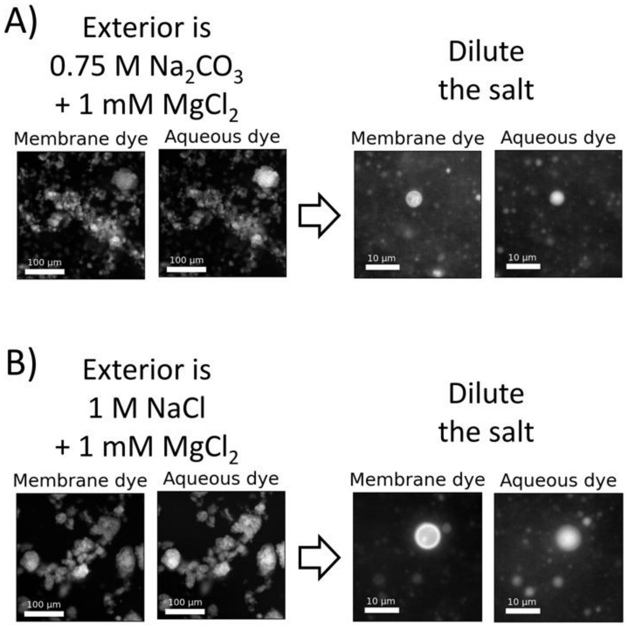


**Fig. S16.** Vesicles of 1:1 decanoic acid: decanol encapsulate aqueous carboxyfluorescein dye for at least 1 hour when the exterior solution is artificial lake water at pH 10. Vesicles were initially assembled in a dilute salt solution of 0.1 M $NaHCO_3$ at pH 10 with 0.1 mM carboxyfluorescein dye before the exterior solution was replaced with artificial lake water. Subsequent dilution into a 56 mM decanoic acid and 0.1 M $NaHCO_3$, pH 10 solution causes vesicles to separate from each other, and separated vesicles still retain encapsulated dye. Rhodamine 6G membrane dye was added immediately prior to imaging. Both sets of experiments were performed at pH 10, and 0.1 M $NaHCO_3$ was included to buffer pH in artificial lake water of 1 M NaCl and 1 mM $MgCl_2$.

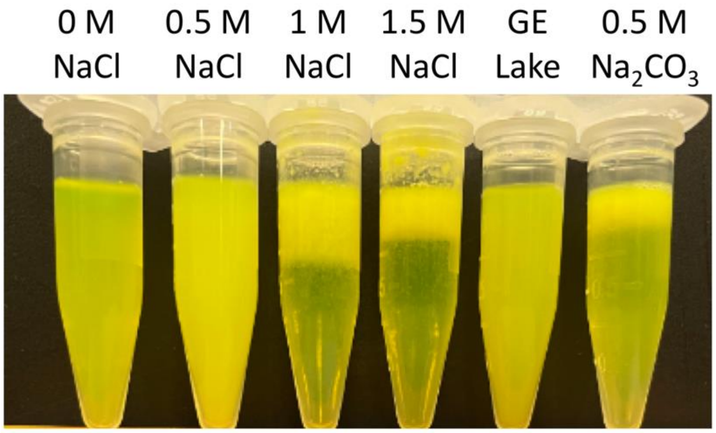


**Fig. S17.** Floatation of flocculated vesicles requires ~1 M unchelated $Na^+$. Because flocculated vesicles do not form a floating layer when the exterior solution is Goodenough Lake (GE), we cannot determine whether vesicles retain dye. Similarly, not all vesicles float when the exterior solution is artificial lake water of 0.5 M $Na_2CO_3$ and 1 mM $MgCl_2$ at pH 10. All samples except natural Goodenough Lake water contain 1 mM $MgCl_2$ at pH 10. NaCl solutions also contain 0.1 M $NaHCO_3$ buffer.

**Section 5: SI References**

1. L. I. Gordon, J. C. Jennings, A. A. Ross, J. M. Krest, "A Suggested Protocol for Continuous Flow Automated Analysis of Seawater Nutrients (Phosphate, Nitrate, Nitrite and Silicic Acid) in the WOCE Hydrographic Program and the Joint Global Ocean Fluxes Study" in (Oregon State University, 1993).

2. J. D. H. Strickland, T. R. Parsons, "A Practical Handbook of Seawater Analysis, 2nd edition." in (Fisheries Research Board of Canada, 1972).

3. S. Haas, K. P. Sinclair, D. C. Catling, Biogeochemical explanations for the world's most phosphate-rich lake, an origin-of-life analog. *Commun Earth Environ* **5**, 1–11 (2024).

4. D. Ding, L. Zhou, C. Giurgiu, J. W. Szostak, Kinetic explanations for the sequence biases observed in the nonenzymatic copying of RNA templates. *Nucleic Acids Research* **50**, 35–45 (2022).
